# Effects of Moxibustion Temperature on Blood Cholesterol Level in a Mice Model of Acute Hyperlipidemia: Role of TRPV1

**DOI:** 10.1155/2013/871704

**Published:** 2013-07-01

**Authors:** Gui-Ying Wang, Ling-Ling Wang, Bin Xu, Jian-Bin Zhang, Jin-Feng Jiang

**Affiliations:** ^1^The Second Clinical Medical College, Nanjing University of Traditional Chinese Medicine, Nanjing 210046, China; ^2^Department of Acupuncture and Moxibustion, Zhongda Hospital Affiliated to Southeast University, Nanjing 210029, China

## Abstract

*Objectives*. To compare the effects of moxibustion at two different temperatures (38°C and 46°C) on the blood cholesterol level in a mice model of acute hyperlipidemia, to detect the different expression levels of transient receptor potential vanilloid subfamily 1 (TRPV1) in the dorsal root ganglions of the wild mice, and to explore the correlation between TRPV1 and moxibustion's cholesterol-lowering effects. *Method*. Two different mice models were used: C57BL/6J wild type (WT) and TRPV1 gene knockout (TRPV1−/−). Each model was randomly divided into control group and model group with three subgroups after acute hyperlipidemia was established: model control group, 38°C moxibustion group, and 46°C moxibustion group. The mice in 38°C group and 46°C group were subject to moxibustion. After the therapy, the cholesterol concentration in serum was measured, and the expression of TRPV1 was quantified. *Results*. In WT mice, moxibustion caused a decrease in blood cholesterol level and upregulation of TRPV1 at the mRNA level, which was significantly greater in the 46°C group. In contrast, in TRPV1−/− mice, the differences of cholesterol-lowering effects of moxibustion were lost. *Conclusions*. Temperature is one of the important factors affecting the effects of moxibustion, and the cholesterol -lowering effect of moxibustion is related to the activation of TRPV1.

## 1. Introduction

Moxibustion is a unique therapeutic method in traditional Chinese medicine (TCM), which has long been used to treat human diseases through stimulating certain regions or sites of lesion on the body surface by applying heat with ignited moxa wool. Investigators analyzed the biophysical features of moxibustion and pointed out that the thermal effect or temperature is one of the important determinants of the therapeutic efficacy of moxibustion [1]. Heat produced by moxibustion stimulates not the only epidermis but also the subcutaneous tissue and muscles. Thermal stimulus is an important factor for the therapeutic effects of moxibustion. Thermal stimulation mainly changes the temperature of the skin around the acupuncture points on the body surface to induce effects.

Normal body temperature is the necessary condition for metabolism and vital movement of the body. The body regulates its temperature by using its periphery and central thermoreceptors. In mammals, ion channels are regarded as the primary thermal signal conductors for thermoreception. Some ion channels can be directly activated by changes of body and skin temperature, as represented by transient receptor potential (TRP), a class of diverse ion channels that play significant roles in regulating a wide spectrum of cellular processes [2]. TRP was firstly described by Cosens and Manning [3] when they recorded transient potential, as opposed to the normal continuous potential, in the related mutants of the drosophila's retina photoreceptor. TRPV family members are nonselective ion channels which are highly relevant to mammals' thermoreception. When TRPV is activated, the inflow of Ca^2+^ is induced. Increased intracellular Ca^2+^ concentration causes changes in physiological and pathophysiological function [4]. TRPV1, TRPV2 (transient receptor potential vanilloid subfamily 2), TRPV3 (transient receptor potential vanilloid subfamily 3), and TRPV4 (transient receptor potential vanilloid subfamily 4), also known as capsaicin receptors and the vanilloid receptors, are major TRPVs that are thermal sensitive. Among these, TRPV1, the most extensively studied TRPV, has been believed to play a key role in response to thermal fluctuations in mice [5]. It is mainly distributed in the dorsal root ganglion neurons, trigeminal ganglion, nodosal ganglion, and spinal and peripheral nerve endings [6]. TRPV1 is not only sensitive to capsaicin but can also be activated by harmful heat challenge (>43°C), acid (pH < 5.90), inflammatory mediators (such as metabolites of arachidonic acid), tissue injuries, changes of extracellular osmolarity, intracellular redox condition, and electrostatic charge, indicating that TRPV1 is a polymodal receptor [7]. TRPV1−/− mice were found less affected or not affected by heat. Blockers increase body temperature in animals and humans, indicating that TRPV1 is a necessary biomolecule in the heat and pain sensation and body temperature regulation [8–10]. Yet, whether TRPV1 is involved in the thermal effect of moxibustion remained unexplored.

In clinical practice, a variety of moxibustions such as moxibustion with seed-sized moxa cone, moxibustion with moxa cone, and moxibustion with moxa stick all can make patients feel “heat.” The special feeling called “feeling of moxibustion” can be presented as “warm, hot, burnt, and painful.” Different ways of moxibustions can produce different temperature changes and activate different thermoreceptors on the skin to induce different biological effects. But does the thermal stimulation or the pain stimulation play the key role in moxibustion? What is the optimal temperature of moxibustion for patients to tolerate yet with the best therapeutic effects? Can moxibustion at different temperatures cause different therapeutic effects? Is there a correlation between the mechanism of moxibustion and the activation of TRPV1? These questions remained yet to be answered.

This study was designed to investigate the potential role of TRPV1 in regulating the thermal dependence of the therapeutic efficacy of moxibustion. The temperature was set at 38°C which is lower than 43°C and 46°C which is higher than 43°C, respectively, as the interference conditions. TRPV1 WT mice and TRPV1−/− mice were used to establish a mouse model of acute hyperlipidemia. Influences of moxa roll moxibustion on the blood cholesterol level and the expression of TRPV1 in the dorsal root ganglions of mice under different temperatures were assessed.

## 2. Materials and Methods

### 2.1. Experimental Materials

#### 2.1.1. Experimental Animals

Two-month-old C57BL/6J WT mice of 18–22 g were purchased from the Comparative Medicine Centre of Yangzhou University (License code: SCLXK (SU)2007-0001) and were maintained in the Experimental Animals Center of Nanjing University of Chinese Medicine (clean). Two-month-old TRPV1−/− mice of 18–22 g were purchased from the Model Animal Research Center of Nanjing University (Certification Code: 0006920) and were maintained in the SPF Experimental Animals Center of Nanjing University of Chinese Medicine. Use of animals was in accordance with the *Guidelines to Treat the Experimental Animals* published by the Ministry of Science and Technology of People's Republic of China [11]. 

**Figure 1:**
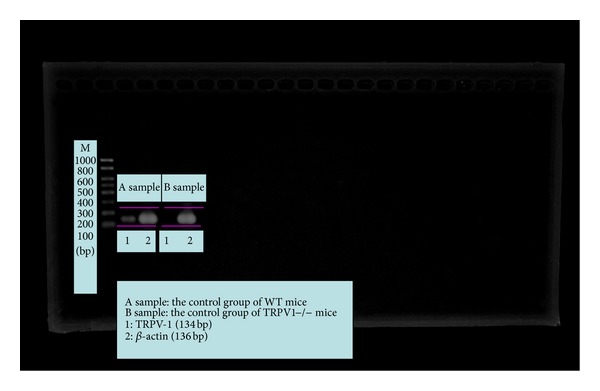
Comparison of electrophoretograms in the control WT mice and the control TRPV1−/− mice.

Figure 1 proved that TRPV1−/− mice in this experiment were successful TRPV1−/− models.

#### 2.1.2. Major Experimental Instruments

The following instruments were utilized in our experiments: moxibustion stick with a diameter of 7 mm (Nanyang Hanyi Moxibustion Technology Development Co., Ltd.), temperature-measuring instrument (HC-04 type, provided by HZNG Zhou Hong Chang Technology Co., Ltd.), analytical balance (Sartorius, Germany, BL310, BL21S), tissue homogenizer (Eppendorf, Haimen, China), whirlpool oscillator (XW-80A, Kylin-Bell, Haimen, China), PCR cycler (Labnet, America MultiGene Gradient), Fluorescence quantitative PCR cycler (DA An Gene Co., Ltd. of Sun Yat-Sen University, DA7600), DNA electrophoresis meter (Liuyi Instrument Company, Beijing, DYY-6B), gel imager (BIO-RAD, America, Gel Doc XR), agent knit of composing the first chain of cDNA (Fermentas, Lithuania, PC0002), operative light microscope (SXP—1C Yiguang Instrument Co., Ltd., Shanghai), and ice maker (Aisinuo, China, GM-100K).

### 2.2. Experimental Methods

#### 2.2.1. Method to Establish Models

The mouse model of acute hyperlipidemia was created according to the procedures described in *Experimental Method in Pharmacology*. Briefly, the egg yolk extracted from fresh eggs was blended with physiological saline to make 75% eggnog. Mice under fasting state for 16 h were weighed and injected with the eggnog at a dose of 0.5 mL per 20 g per mouse. After 20 h, the mice were used for experimental measurements [12].

#### 2.2.2. Subgroups

A total of 32 C57BL/6J WT mice of 2-month age were divided into control (*n* = 8) and model (*n* = 24) groups. The mice of the model group were randomly subdivided into the model control, 38°C moxibustion, and 46°C moxibustion subgroups with 8 mice in each category. Similarly, a total of 24 TRPV1−/− mice were first divided into control (*n* = 6) and model (*n* = 18) groups. The TRPV1−/− mice of the model group were further divided into model control, 38°C moxibustion, and 46°C moxibustion subgroups with 6 mice in each category.

#### 2.2.3. Acupuncture Points

To fix the acupuncture points we took human as the model: Shenque acupuncture point was located in the juncture formed by the margo superior of the manubrium sterni along the ventral median line of the mice and the upper 3/4 and lower 1/4 of external genital organs [13]; Zusanli acupuncture point was located in the posterior and lateral part of knee joint 5 mm under the capitulum fibulae [14]. 

#### 2.2.4. Temperature-Setting Preexperiment

**Figure 2:**
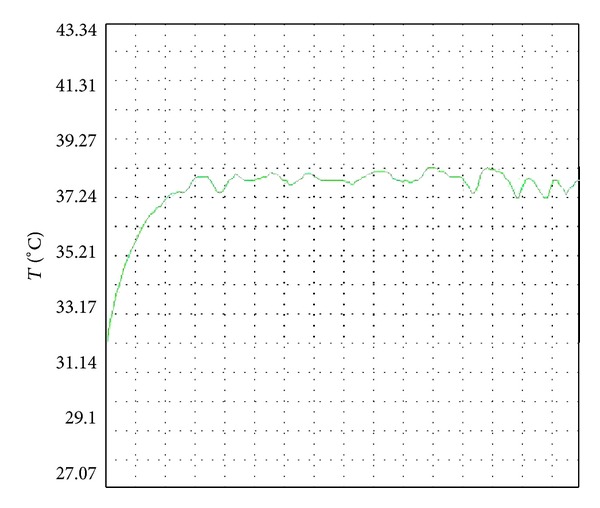
Comparison of temperature curves on the local skin during moxibustion at 38°C.

**Figure 3:**
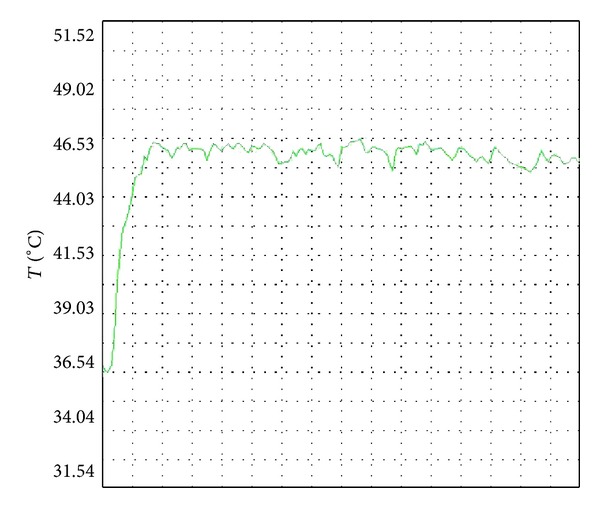
Comparison of temperature curves on the local skin during moxibustion at 46°C.

A preexperiment was performed before the formal experiment at under 26°C. Hair located 1 cm × 2 cm around the acupuncture points of the normal WT mice was cut off to expose local skin which was then disinfected by alcohol tampon. Mice were fixed in the dorsal decubitus position by self-made fixation clamps. Animal-use moxa sticks with the diameter of 7 mm and length of 20 cm were burned to operate moxibustion directly on the acupuncture points. The probe of temperature-control instrument was put on the skin where acupuncture points were located to detect instantaneous temperature. Temperature was controlled by adjusting the distance between moxa sticks and the skin, blowing the fire of the stick, and adjusting the frequency of flipping the ash of moxa stick. These steps were performed repeatedly until the temperature was stabilized, and then the distance between the moxa stick and the skin where acupuncture points were located was measured. When the distance was 35 ± 5 mm, the temperature was stabilized at 38°C ± 1°C; when the distance was 10 ± 2 mm the temperature was stabilized at 46°C ± 1°C. Figures 2 and 3, respectively, show the temperature curves on the local skin during moxibustion at 38°C and 46°C.

#### 2.2.5. Treatments

Treatments were performed at the same day when models were established. Room temperature was fixed at 26°C. Hair located 1 cm × 2 cm around the acupuncture points of the mice was cut off to expose the skin which was then disinfected by alcohol tampon. Mice were fixed in the dorsal decubitus position by self-made fixation clamps. After the mice had become quiet without struggling, moxibustion was performed. The method to control the temperature was the same as in the preexperiment. Moxibustion was performed in both Shenque and double Zusanli points for 10 min per point per day for 2 days in total. During the treatment, mice in the control and the model control groups were sham-treated without moxibustion. 

**Figure 4:**
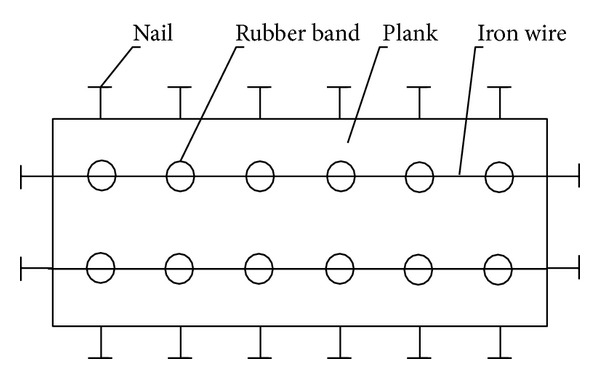
A plane view of the self-made fixation clamp.

Usage of the self-made fixation clamp is shown in Figure 4. Fix the mouse in supine position on the plank, and fix the limbs through the rubber ring; strain the four jaws with the rubber band to prevent the mouse from moving; then, fix the rubber band onto the adjacent nail.

#### 2.2.6. Materials

After moxibustion treatment, blood samples were taken by excising the eyeballs. Serum was extracted by centrifuging at 3000 r/min for 15 min. The supernatant was collected into a 0.5 mL Eppendorf tube and then stored at −20°C for later uses. 

After sacrificing, the back skin of the mice was cut open along the spine which was separated on ice bath. Canalis spinalis was cut open along the abdominal side of the spine. Nerve fibers were cut and stripped. Under a light microscope, dorsal root ganglions at lumbar segments were dissected from apertura spinalis by microforceps. Residual nerve fibers were ticked out. Dorsal root ganglions were placed into a tube and frozen in liquid nitrogen for later uses.

#### 2.2.7. Serum Testing

 Serum samples were sent to Biochemical Room of Clinical Laboratory of Zhongda Hospital Southeast University where the serum cholesterol level was determined by oxidase reaction. 

#### 2.2.8. Real-Time PCR

Sequence of the primer and the products of PCR: Mus-*β*-actin primer (136 bp): Forward primer: 5-GCAGAAGGAGATTACTGCTCT-3 Reverse prim 5-GCTGATCCACATCTGCTGGAA-3 Mus-TRPV-1: primer (134 bp): Forward (oIMR1561)primer: 5-CCTGCTCAACATGCTCATTG-3 Reverse (oIMR1562)primer: 5- TCCTCATGCACTTCAGGAAA-3.

After extracting RNA, the purity and concentration of RNA were measured. The first-strand cDNA was synthesized by reverse transcription. Real-time PCR amplification was performed with each sample in triplicate. The reaction reagents included 2X PCR Master Mix (SYBR Green) 10 *μ*L, 1 *μ*L template (cDNA dilated for 10 times), 2 *μ*L primer MIX (F/R 10 *μ*M, resp.), and DEPC water 7 *μ*L, 20 *μ*L. PCR products were quantified by the 2-ΔΔCT method.

#### 2.2.9. Statistical Analysis

SPSS17.0 statistical software was adopted to analyze the data. All data are presented as $\overset{-}{x}\pm s$ and processed by one-factor analysis of variance. LSD method was used to compare data between groups. 

## 3. Results

### 3.1. Comparisons of Serum Cholesterol in WT C57BL/6J Mice

Compared with the mice in the control group, the mice in the model control group showed significantly increased serum cholesterol level (*P* < 0.01), indicating that the model of acute hyperlipidemia was successfully established. In contrast to the mice in the model control group, mice subjected to moxibustion at 38°C and at 46°C both demonstrated decreased serum cholesterol levels; only the difference between the model control group and 46°C moxibustion group reached statistical significance (*P* < 0.001). Notably, the blood cholesterol drop was to a significantly greater extent in animals subject to 46°C moxibustion than those with 38°C moxibustion (Table 1).

### 3.2. Serum Cholesterol Levels in TRPV1−/− Mice

Like the WT animals, the TRPV1−/− mice with acute hyperlipidemia had significantly higher serum cholesterol concentration (*P* < 0.01). The serum cholesterol concentration was not significantly altered by moxibustion at either 38°C or 46°C (*P* > 0.05). There was no significant difference between 38°C moxibustion and 46°C moxibustion (Table 2).

### 3.3. mRNA Levels of TRPV1 in the Dorsal Root Ganglions of C57BL/6J WT Mice

**Figure 5:**
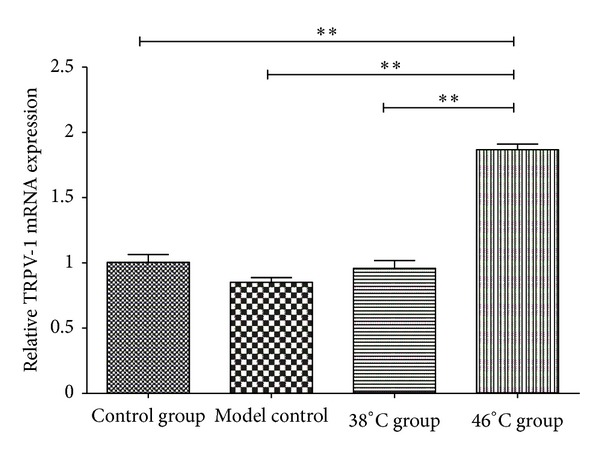
Relative TRPV-1 mRNA levels in C57BL/6J WT mice.

Figure 5 demonstrated that the relative TRPV-1 mRNA expression in the moxibustion at 46°C group differed significantly from the control group (*P* < 0.01); the relative TRPV-1 mRNA expression in the moxibustion at 46°C group differed significantly from the model control group (**P < 0.01). Differences between the moxibustion at 46°C group and the moxibustion at 38°C were significant (**P < 0.01), indicating that the temperature of moxibustion was an important factor affecting TRPV1 mRNA expression in the dorsal root ganglions of mice.

### 3.4. PCR Experiment Results of Dorsal Root Ganglions of TRPV1−/− Mice

**Figure 6:**
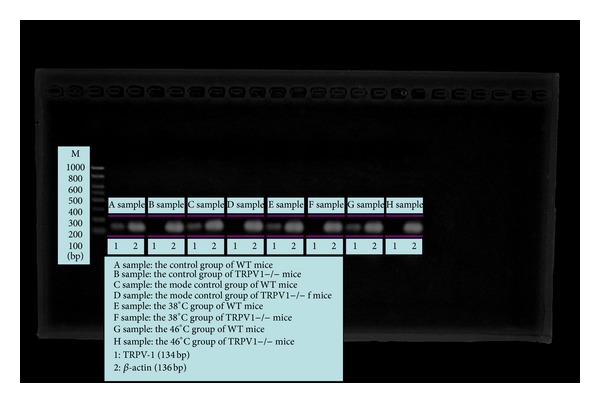
Electrophoretograms of four PCR experiments on WT mice and TRPV1−/− mice.

Figure 6 proved again that TRPV1 in the TRPV1−/− mice has been successfully knocked out. 

## 4. Discussions

Moxibustion can stimulate the human body's regulating system to prevent and treat a disease by producing thermal effects on the specific position to stimulate thermoreceptors on the skin. Moxibustion has had a long history, and its therapeutic mechanisms won wide attentions. Ancient Chinese doctors had already noticed the importance of moxibustion's thermal effects. *Treatise on the Appropriateness of Different Methods according to Locality *concluded that “coldness in organs can cause many diseases and these diseases should be treated with moxibustion.” Wang Bing, a renowned ancient Chinese doctor, said “moxibustion meant to burn moxa to treat diseases,” indicating that burning was the key element for moxibustion. Recent studies have pointed to thermal stimulation as the major factor of moxibustion, and heat produced by burning was the key in therapy [15, 16]. 

*Lingshu Cijiezhenxie* concluded “smooth running of fire and Qi will make blood and body fluid flow.” *Wai Tai Mi Yao* introduced “if the temperature at which moxibustion is performed to the patient is not high enough, the patient's moxibustion feeling will not be strong enough to cure the disease.” Medical scholars in ancient China emphasized “heat” which is also called “moxibustion feeling” and pointed out that therapeutic effects of moxibustion would only show when the fire and Qi in the body were running smoothly which took heat at the right temperature as a prerequisite [17]. Modern acupuncturists also paid attention to the relationship between the temperature of moxibustion and its therapeutic effects. For example, Ling et al. [18] observed, with moxibustion under different temperatures and on different skin regions, the activation of subnucleus reticularis dorsalis (SRD) at dorsal segment of rats' medulla oblongata. According to their study, moxibustion under 40°C or 42°C could not activate SRD on any area; above 46°C, the area stimulated by moxibustion showed linear correlation with the discharging activity of SRD and under the same temperature, and discharging activity of SRD did not increase with the enlargement of stimulated area. Xinmin et al. [19] observed the relationship between the pyretolysis effects of moxibustion at different temperatures and the activities of temperature-sensing neurons in the thermotaxic center. According to their study, moxibustion under 48°C showed significant rivalry effects against endotoxin (ET) and discharging effects of heat-sensitive neuron (HSN); moxibustion at 40°C showed no obvious effects. Xinmin et al. thought that moxibustion under different temperatures might activate different thermoreceptors on the skin.

The present study revealed the relationship between the temperature of moxibustion and its therapeutic effects. During the repeated preexperiments in early periods, we controlled the moxibustion temperatures at 38°C ± 1°C and 46°C ± 1°C on the targeted skin areas by using HC-04 temperature-measuring instrument. A temperature value of 46°C is considered the thermal threshold that the human body can tolerate or feel the pain of being slightly burned while 38°C is the temperature that is a little higher than the body temperature of humans and makes humans feel comfortable. 

In this study, WT mice in the 46°C moxibustion group showed a stronger cholesterol-lowering effect than those in the 38°C moxibustion group, supporting the notion that temperature is an important factor in determining the therapeutic effects of moxibustion. Many clinical reports [20, 21] also put forward that warm feeling on the skin area where moxibustion is performed can never cure diseases; it is widely accepted that burning feeling is necessary to generate beneficial effects with moxibustion; burning feeling produced by thermal stimulation is induced by the activation of skin receptors. This “burning feeling” is regarded as a good sign for the appropriate strength of moxibustion. During moxibustion, simulating certain regions on the body by applying heat with ignited moxa wool is the key procedure. Therefore, in clinical practice temperature setting for moxibustion should always be seriously considered. 

We selected two different mice models, because TRPV1−/− mice is a successful model based on C57BL/6J WT mice, the two type of mice were the same species, that is why they can be used to control study of TRPV1. Our results also showed that in WT animals after moxa roll moxibustion at different temperatures, the upregulation of TRPV1 expression in dorsal root ganglions was considerably greater in the 46°C moxibustion group than in the 38°C moxibustion group. Similarly, the cholesterol-lowering effect in the mice with acute hyperlipidemia was also found to be stronger with 46°C moxibustion than with 38°C moxibustion. In TRPV1−/− mice, however, no significant differences in cholesterol level were observed with moxibustions either at 46°C or 38°C or between these two temperatures. These findings indicate that TRPV1 is an important molecular regulator that confers the temperature dependence of the cholesterol-lowering effects of moxibustion. And there was a relationship between the starting mechanism of moxibustion's cholesterol-regulating mechanism and the activation of TRPV1.

Thermal effects are important for moxibustion's therapeutic effects. The heat can stimulate many kinds of skin receptors and influence the biochemical metabolism of nervous system and histiocytes. Some investigators have recorded the discharging activities of temperature-sensing nerve fibers [22]. They found that sensory neurons express proteins that can transmit temperature and can be activated by specific temperature changes to work as molecular temperature detectors. Thermal stimulation of moxibustion is in fact an energy-transmitting process, and TRP family may be the primary transducer for this process. TRPV1 is a thermoreceptor and can be activated at the temperature above 43°C. Moxibustion at 46°C can activate TRPV1 and induce the inflow of Ca^2+^ which can cause the muscle fibers to act. Production of action potential will be transmitted to the central nervous system to start moxibustion effect.

Thus, manifestation of moxibustion effect is correlated with the activation of TRPV1 channels. The optimal temperature for tolerable moxibustion with the best therapeutic effects should be a threshold higher than 43°C. considering that the sample size was statistically significant, we chose this quantity of mice in each group. The larger the sample size is, the more convincing the study is. So we will consider it for the experimental design in the future. To understand how the thermal stimulation during moxibustion initiates the signaling cascade and to translate the signal into moxibustion effects and whether other TRP family members are also involved in moxibustion effects, further studies are needed.

## Figures and Tables

**Table 1:** Comparison of serum cholesterol level of C57BL/6J WT mice in each group ($\overset{-}{x}\pm s$).

Groups	Cholesterol (mmol/L)	*F*	*P*
Control group	2.17 ± 0.46	25.31	0.000
Model control group	5.24 ± 0.76^#^		
38°C group	4.41 ± 1.21		
46°C group	2.44 ± 0.29^▲☆^		

Notice. ^#^ *P* < 0.001 (*P* = 0.000) versus control; ^▲^ *P* < 0.001 (*P* = 0.000) versus model control; ^*☆*^ *P* < 0.01 (*P* = 0.003) versus moxibustion 38°C.

**Table 2:** Serum cholesterol level in TRPV1−/− mice ($\overset{-}{x}\pm s$).

Groups	Cholesterol (mmol/L)	*F*	*P*
Control group	3.66 ± 0.42		
Model control group	6.00 ± 1.26^#^	3.30	0.04
38°C group	4.96 ± 0.62		
46°C group	4.58 ± 2.16		

Notice. ^#^ *P* < 0.01 (*P* = 0.006) versus control.
